# C1–2 facet disarticulation for correction of iatrogenic cervical kyphosis following occipital-cervical fusion

**DOI:** 10.3171/2020.4.FocusVid.20175

**Published:** 2020-07-01

**Authors:** Miki Katzir, Aboubakr T. Amer, Asad S. Akhter, Stephanus V. Viljoen, Ehud Mendel

**Affiliations:** Department of Neurological Surgery and the James Cancer Hospital, Wexner Medical Center, The Ohio State University, Columbus, Ohio

**Keywords:** occipital-cervical fusion, degenerative cervical spine, occiput–C2 angle

## Abstract

The patient is a 69-year-old woman with a history of atlantoaxial instability and cervical pain who underwent an occipital-cervical fusion at an outside hospital. Five days following the procedure she required a PEG tube due to progressive dysphagia. Compared with preoperative imaging, x-ray shows cervical spine hyperextension with a significant decrease in the occipital–C2 angle. A swallow test confirmed aspiration and pharyngeal phase functional impairment. Two-stage surgery consisted of hardware removal, drilling the fused right C1–2 facet, reinstrumentation, and halo placement. The swallowing test confirmed there is no aspiration. We proceeded with rod placement. The patient recovered completely.

The video can be found here: https://youtu.be/YzdJrOm46Y4

## Transcript

**0:21** This is a case describing a patient undergoing C1–2 disarticulation for correction of iatrogenic cervical kyphosis following occipital-cervical fusion.

**0:31** This is a 70-year-old female with a history of breast cancer status post bilateral mastectomy and chemotherapy. She underwent occipital-cervical fusion at an outside facility for atlantoaxial instability and neck pain refractory to conservative therapy. This was done 7 months prior to presentation to our hospital. Following her initial fixation, she developed severe dysphagia ultimately requiring a PEG tube on postoperative day 5.

**0:57** On physical exam, patient was hyperextended at the neck. Rest of the physical exam was unremarkable. As mentioned previously, she did have significant dysphagia.

**1:09** Pre- and postoperative lateral cervical x-rays from the outside facility demonstrate the change in alignment due to the occipital-cervical fusion shown by the O–C2 angle, which is the Cobb angle referenced by the hard palate and inferior endplate of C2, which has been shown to correlate with postoperative destiny to develop dysphagia. Preoperative angle seen here is 27. Postoperative is 3. This significant difference can explain dysphagia, which is further demonstrated in a modified barium swallow (Izeki et al., 2014; Yoshida et al., 2007) (Fig. 1).

**1:44** Preoperative barium swallow shows laryngeal penetration and aspiration of liquids and functional impairments of the pharyngeal phase. There is also a narrow appearance of the cervical esophageal lumen.

**1:58** Sagittal, axial, and coronal CT shows a C1–2 pannus formation fusing the joint causing cervical canal stenosis at that level (Fig. 2).

**2:08** We offered a staged approach that composed of first removing prior hardware, new instrumentation, placement of occipital plate, C1–4 screws, and drilling the right C1–2 joint in order to mobilize it, and then placing the patient in halo traction in order to perform swallow test before the second stage, which involved final fixation and placement of bilateral C1–2 facet shims (Tang et al., 2019).

**2:37** You can see the C1 posterior arch, C2 lamina, C2 par, C1 lateral mass, and the C1–2 joint.

**2:59** In this model we can easily see the C1–2 joint that was drilled out in order to mobilize it (Fig. 3).

**3:06** Oblique view showing the relationship of the neurovascular structures composed of the vertebral artery and C2 nerve root to the C1–2 joint. These structures must be protected with instruments during the disarticulation process (Fig. 4).

**3:23** You can see the C1 lateral mass. We insert a Penfield 4 medial to the C1 lateral mass to protect the spinal cord throughout the disarticulation. We insert the second Penfield lateral to the C1 lateral mass to protect the vertebral artery.

**3:57** Here we show the intraoperative view of the surgical field. Key anatomical landmarks are labeled (Fig. 5).

**4:11** Here we are drilling out the fused C1–2 joint space using a matchstick drill.

**4:21** With the help of a small osteotome, we completed the release of the C1–2 joint.

**4:27** Positioning was confirmed with intraoperative fluoroscopy (Fig. 6).

**4:34** Mobilization of the previously fused C1–2 joint space can be seen here.

**4:43** We then proceeded to place the occipital plate and C1–4 screws. The patient was also placed in halo traction in order to assess swallowing function before final fixation (Fig. 7).

**4:56** This is the barium swallow following the first stage of the surgery which shows no laryngeal aspiration.

**5:05** On the left, lateral x-rays in the neutral position prior to the patient’s first surgery. The middle image shows alignment after the first surgery. The right image shows postoperative images from our surgery. The O–C2 angles are labeled in each film. You can see that after the patient’s first fixation, there was a significant reduction of the O–C2 angle, which potentially led to her pharyngeal narrowing. Our films show improvement of this angle (Morizane et al., 2018; Sheshadri et al., 2017) (Fig. 8).

**5:40** Follow-up shows intact hardware and maintenance of the alignment, with resolution of dysphagia. She was neurologically intact and had minimal pain (Fig. 9).

**5:52** Here we show side-by-side pre- and postoperative modified barium swallow tests. As noted, there is significant improvement in laryngeal and oral mobility without aspiration.

## Supplemental Information

Supplemental Figures
