# Supplementary material for: C1–2 facet disarticulation for correction of iatrogenic cervical kyphosis following occipital-cervical fusion
Source: Neurosurg Focus Video. 2020 Jul 1;3(1):V5. doi: 10.3171/2020.4.FocusVid.20175 (PMC9542231; doi:10.3171/2020.4.FocusVid.20175)
Supplement: Supplemental Figures [file 20175_supplementalfile.pdf]

NEUROSURGICAL FOCUS VIDEO

ONLINE ONLY

Supplemental material

C1–2 facet disarticulation for correction of iatrogenic cervical kyphosis following occipital-cervical fusion

Miki Katzir, MD et al.

https://thejns.org/doi/abs/10.3171/2020.4.FOCUS20175

DISCLAIMER The *Journal of Neurosurgery* acknowledges that the following section is published verbatim as submitted by the authors and did not go through either the *Journal's* peer-review or editing process.

© 2020 The authors, CC BY 4.0 (http://creativecommons.org/licenses/by/4.0/)

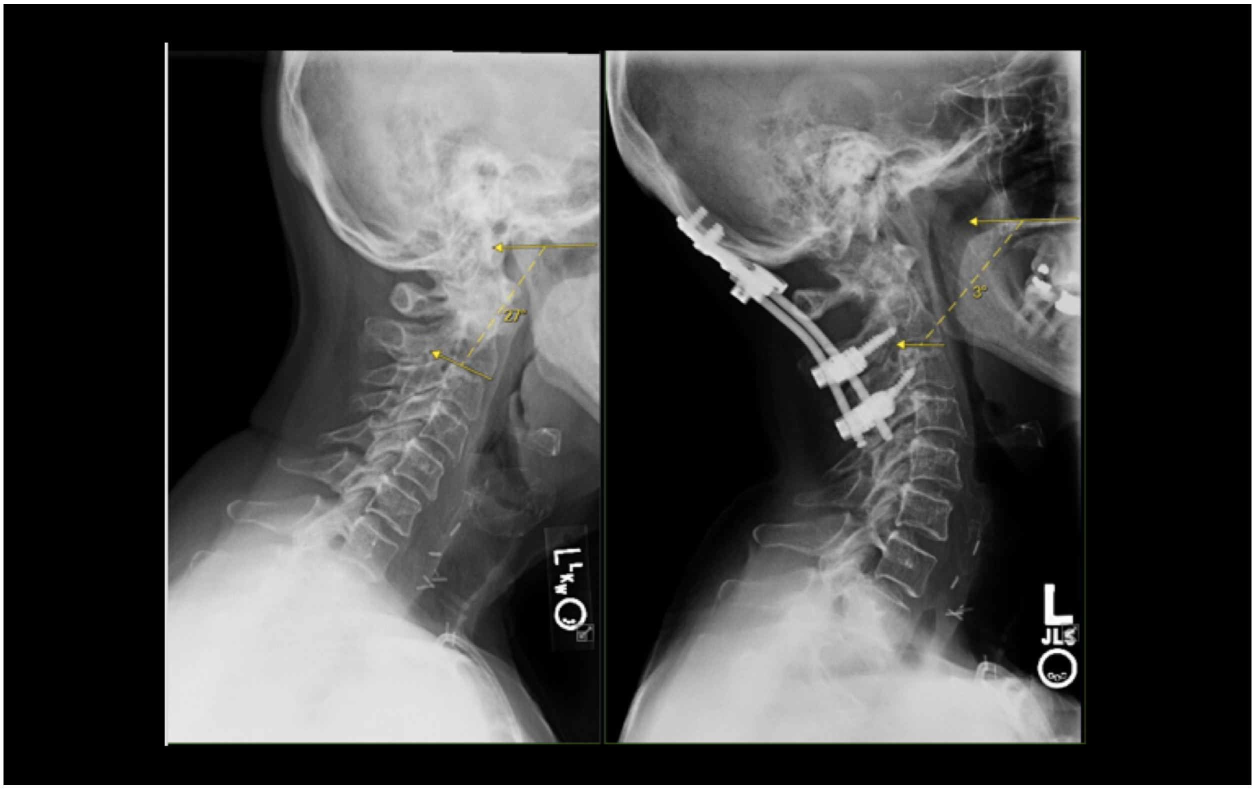

Figure 1. Pre- and post-operative O-C2A. Images obtained from outside facility during initial presentation to our institute.

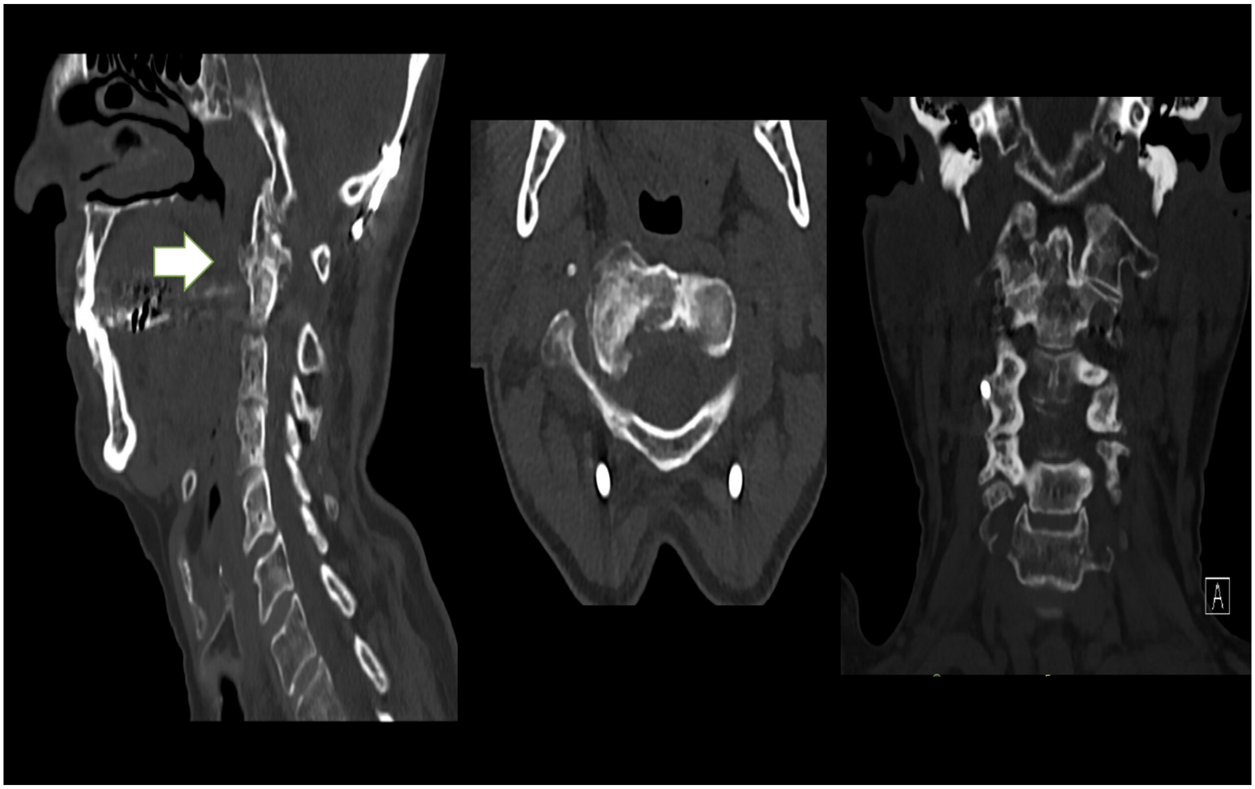

Figure 2. Left, sagittal CT-scan demonstrating pannus formation and hypertrophy at the right C1-C2 joint. Also demonstrated on the axial (middle) and coronal (right) CT scans.

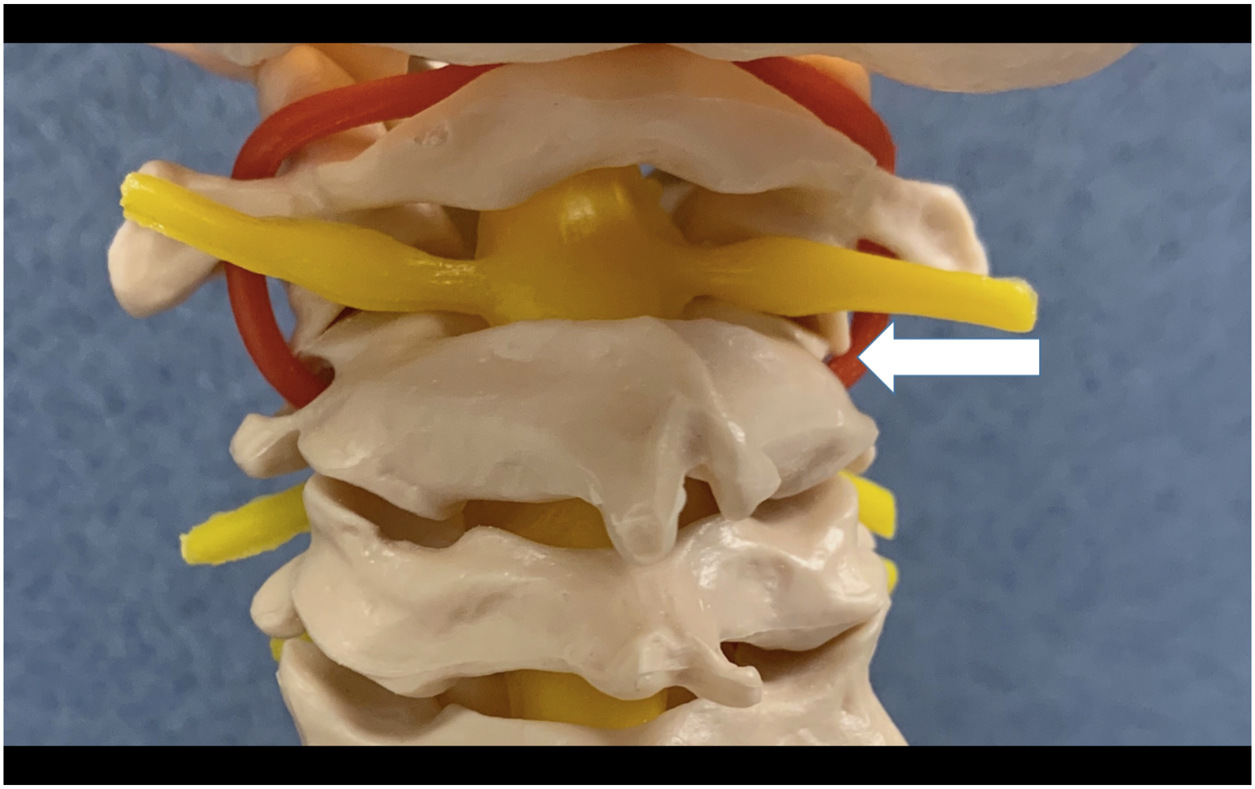

Figure 3. Model view demonstrating the area of the C1-C2 joint in which we drilled.

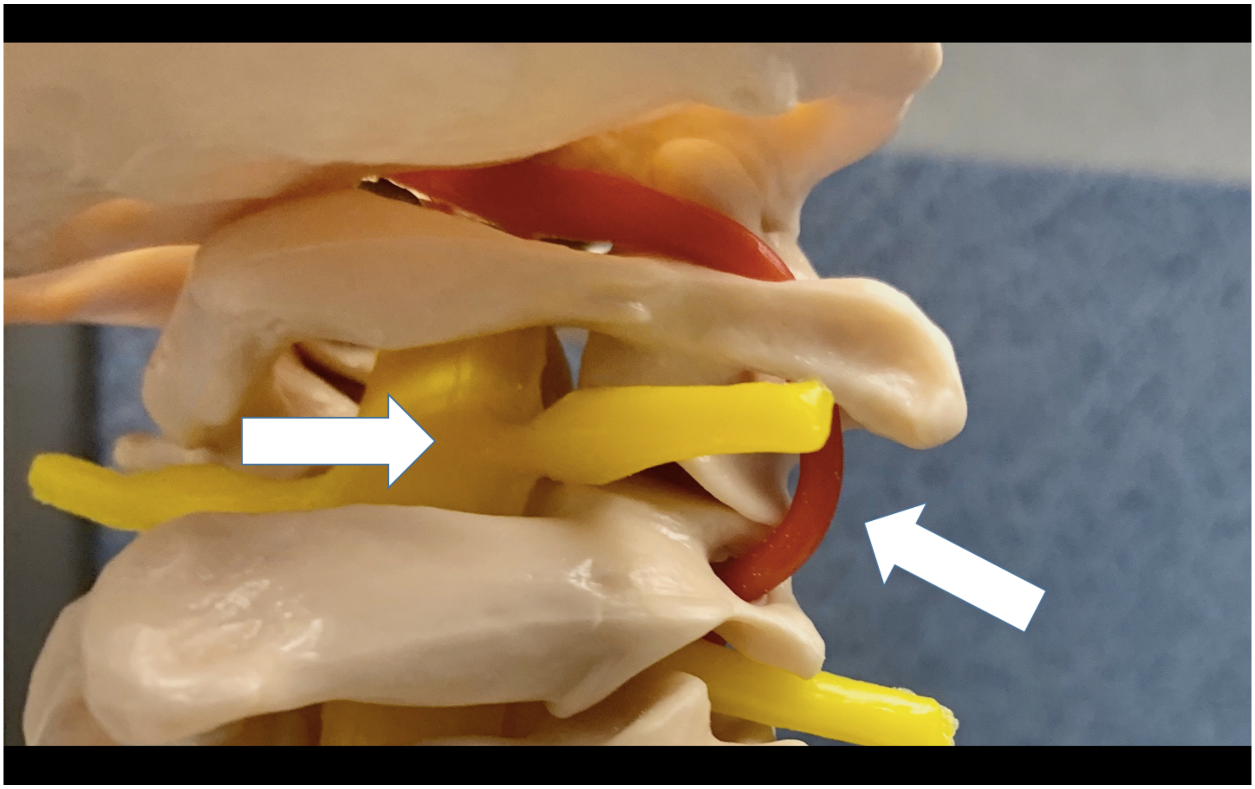

Figure 4. Oblique view of model as seen in Figure 3. Note the relationship of the joint to the spinal cord medially, and the vertebral artery anterolaterally. These structures must be protected during disarticulation.

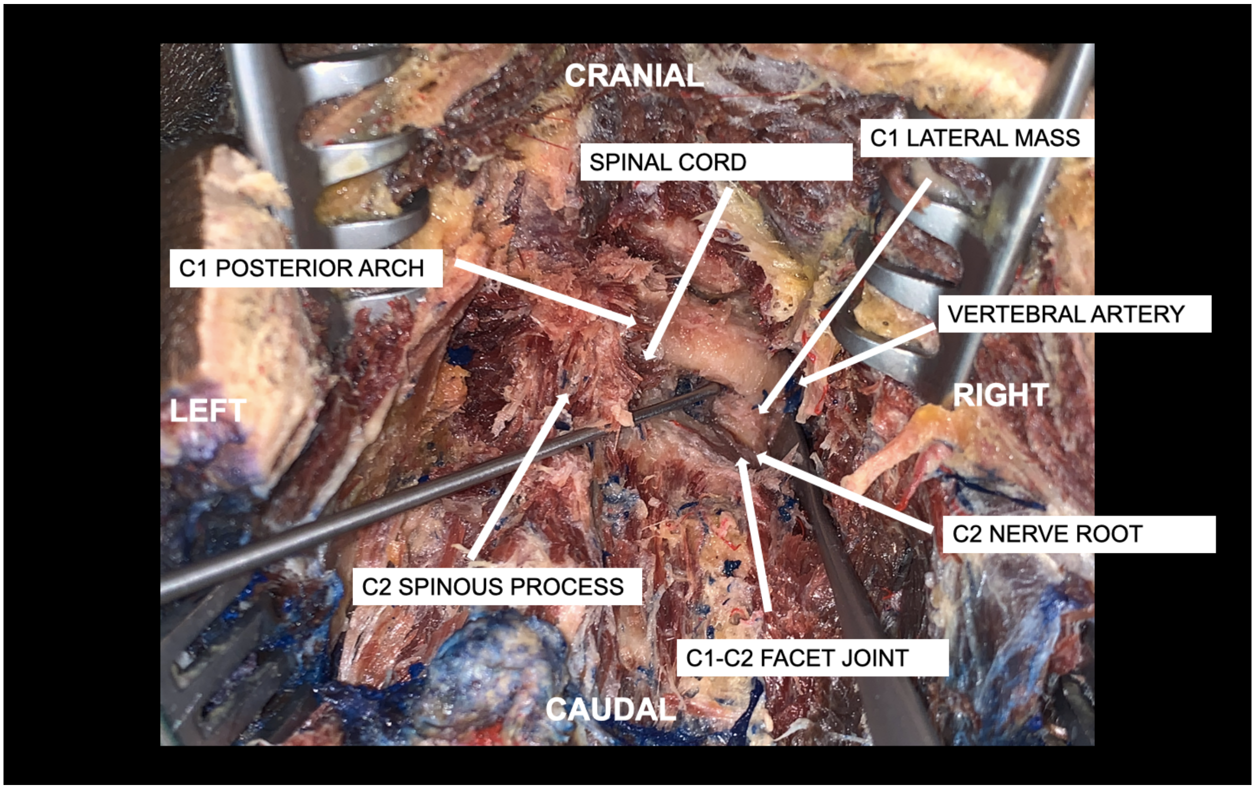

Figure 5. Cadaveric dissection done in our lab with appropriately labeled key anatomical landmarks.

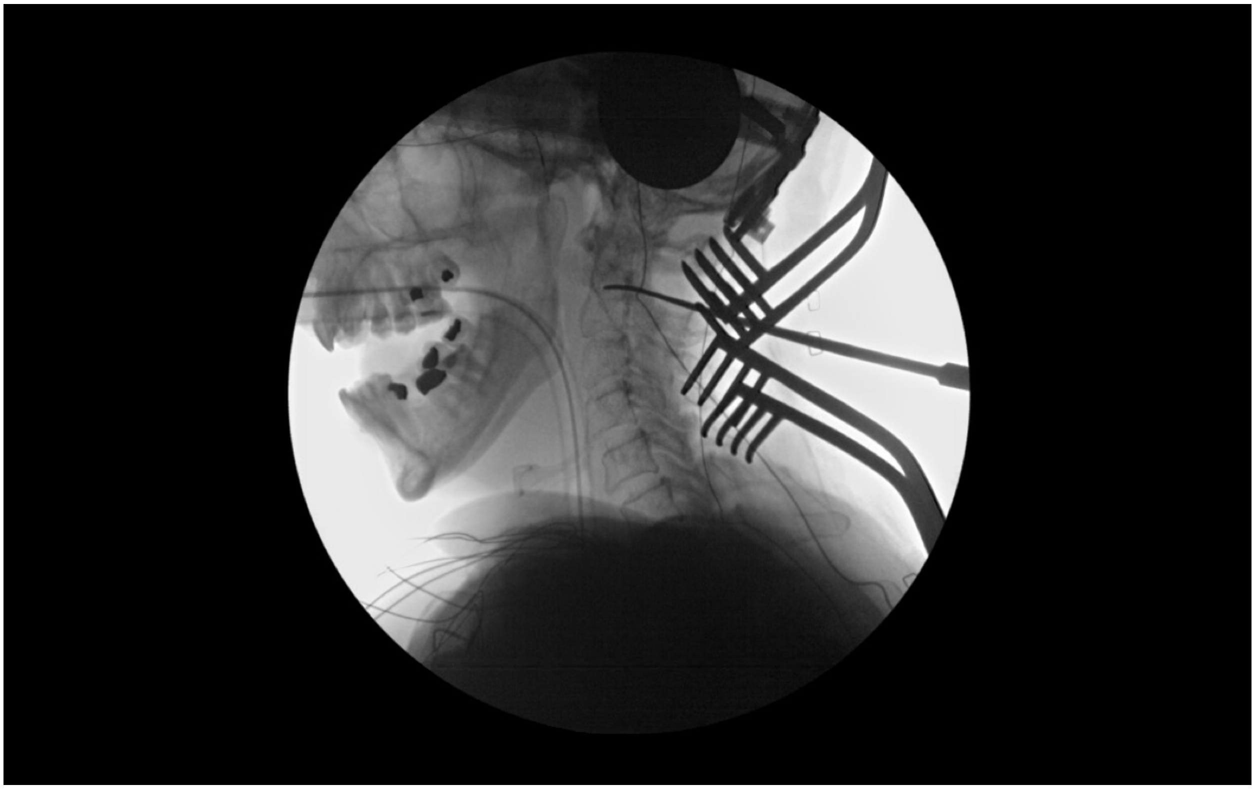

Figure 6. Intra-operative fluoroscopy demonstrating instrument placement verification within the C1-C2 joint space.

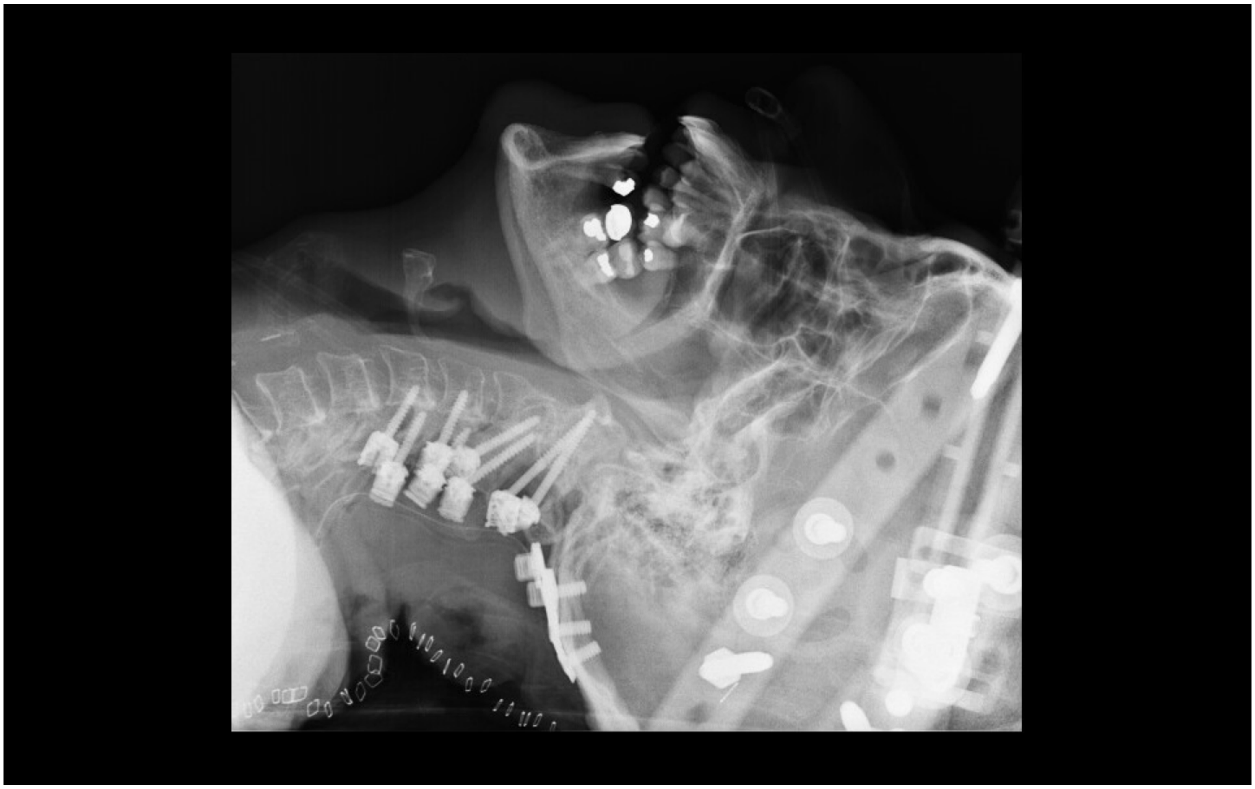

Figure 7. Lateral X-ray film after stage I of surgery demonstrating placement of C1-C4 screws, placement of new occipital plate, and placement of patient in crown halo traction.

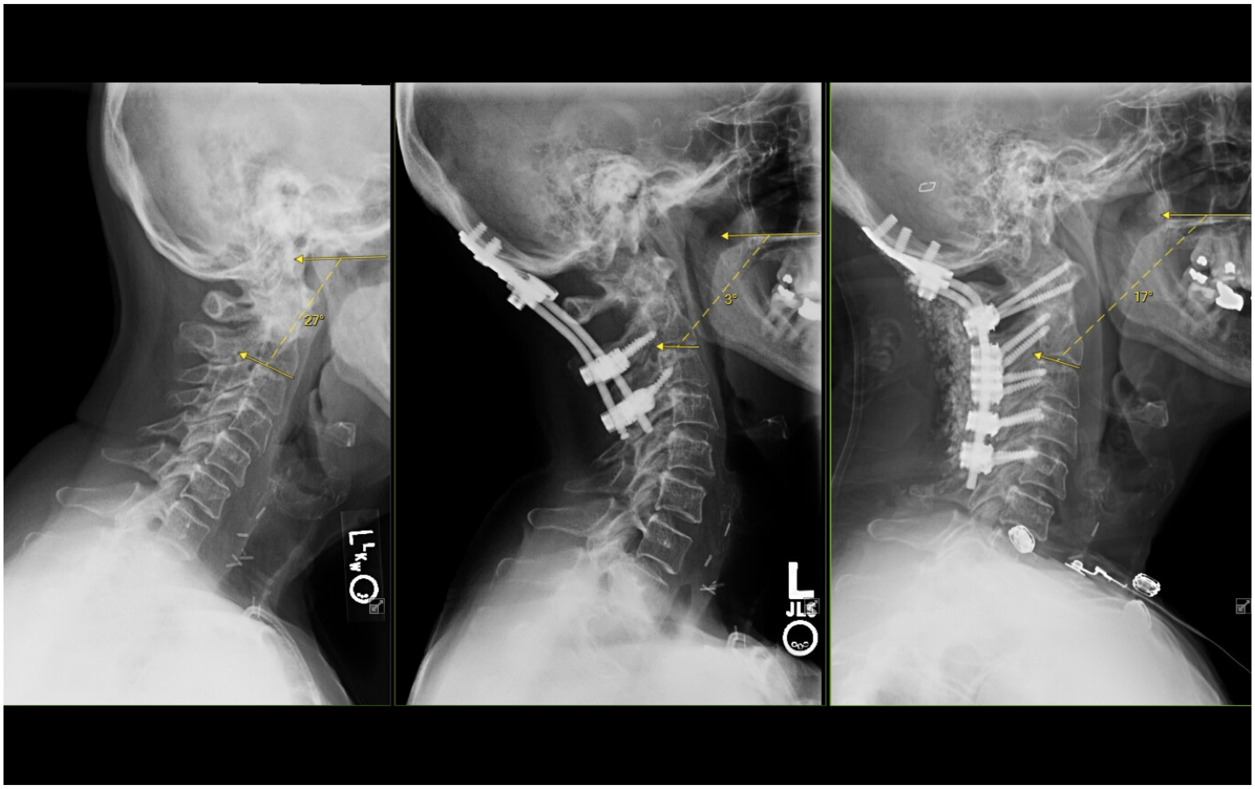

Figure 8. On the left, patient’s lateral films before presentation to the initial outside facility. Middle, lateral films on presentation to our institution. Right, post-operative lateral films after re-instrumentation and disarticulation. Note the initial narrowing of the O-C2A as well as resolution in the post-operative films on the right. It is likely that the patient’s initial post-operative dysphagia as well as her resolution was related to these changes.

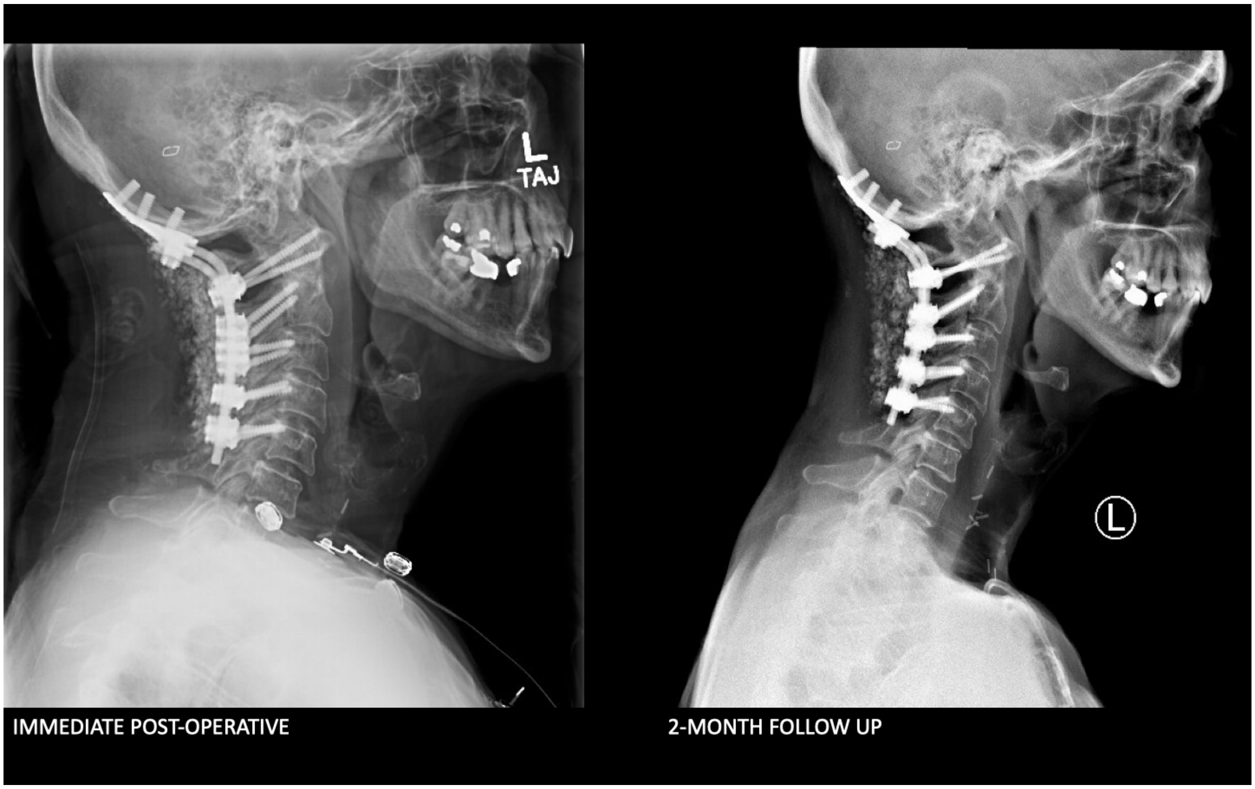

Figure 9. Post-operative lateral x-ray films demonstrating stable hardware and intact alignment.
